# Communication Matters: Exploring the Intersection of Family and Practitioner End of Life Communication

**DOI:** 10.3390/bs7010015

**Published:** 2017-03-19

**Authors:** Leah M. Omilion-Hodges, Nathan M. Swords

**Affiliations:** School of Communication, Western Michigan University, 1903 W Michigan Ave, Kalamazoo, MI 49007-5318, USA; Nathan.M.Swords@wmich.edu

**Keywords:** end of life communication, palliative care, contemporary approaches to end of life, dialectic tensions

## Abstract

After establishing a baseline understanding of some of the factors that influence and shape family end of life communication, empirical research centered on the communication tendencies of nationally-recognized palliative care clinicians is presented. Because death is no longer confined to the bedroom and individuals are increasingly turning to hospitals and health care institutes to assist with end of life, the role of palliative care practitioners is vital. To that end, common communication-rooted issues that may transpire among various medical personnel are explored. Focus on a shared underlying tension—care vs. cure—links the findings between family and palliative care clinician communication regarding end of life. Practical communication solutions and suggestions are offered to facilitate productive and mindful end of life communication between and among family members and health care practitioners.

## 1. Introduction

Oftentimes family communication about end of life (EOL) does not occur until circumstances force loved ones to have these conversations. Moreover, because families are not always preemptive in talking about the death and dying process coupled with the shift of where dying is occurring, these conversations are often taking place within hospitals and health care institutes. As such, health care professionals—commonly palliative care practitioners—become active participants and even leaders in facilitating family communication regarding EOL. Palliative care is patient-centered care, comfort, and support for individuals with chronic and terminal illness and is available to people at any age and at any state of illness unlike hospice.

The current research explores the intersection of palliative care practitioners’ interpersonal and interprofessional communication and the impact practitioners’ communication tendencies may have on families’ EOL communication. More specifically, we focus on how contemporary end of life practices, such as the transition from death at home to death in the organizational setting, is prompting changes such as communal coping where patients, family members, and healthcare personnel collaborate to make EOL decisions.

Through previous work with nationally-recognized palliative care practitioners [1,2] and more current work with use of metaphors and euphemistic language to bridge the topic of death, we have found a common tension. The stress coiled between cure and care continues to complicate family end of life conversations much in the same way it can hinder the interprofessional relationships between medical professionals. The former, cure, aligns with the traditional biomedical approach to medicine that focuses on diagnosing and treating and largely advocates for prolonging life at all costs. This approach has been criticized for treating diseases rather than patients, where a lower quality of life or abatement of one’s wishes may accompany the continued extension of life. Care, the latter, aligns with the more contemporary biosocial model that promotes a patient-centered approach inclusive of a patient’s physical, emotional, spiritual, and psychological needs.

In this essay, we draw from two original data sets to trace the evolution of society’s perceptions of death and how this transition impacts patients, their family members, and health care professionals. In this sense, communication becomes the vehicle through which we make sense of end of life, express or withhold our desires, and influence others whether we are a patient, a family member, or a clinician. Ultimately we leave readers with concrete, communication-rooted suggestions for initiating EOL conversations and for reducing the stigma often part and parcel with the subject. However, we begin by teasing out differences between traditional and contemporary approaches to death and dying and by showing how current practices impact family and practitioner conversations about EOL.

## 2. Contemporary Approaches to Death and Dying 

Although death is a natural process, Western cultures have come to understand the end of life process as something we should avoid and privatize, particularly in the United States. Roughly 63% of Americans die while in hospitals, while an additional 17% die in other institutional settings such as hospice or palliative care [3]. The sustained shift from death at home to death in institutional settings has prompted changes within health care organizations. One transformation is the continued upsurge in palliative care (PC) programs.

PC delivers holistic care addressing patient physical, psychological, emotional, practical and spiritual needs at end of life or in concert with curative treatment. This progressive medical specialty employs individualized communication to provide relief and help alleviate the stress or confusion that may be associated with medical procedures in addition to helping mitigate family dynamics when necessary. Similarly, PC practitioners embrace patients’ families as part of their charge and therefore, often become integral components of family EOL communication. When we consider the change of where dying occurs, it begs the question of how the location change influences EOL communication. Thus in many ways, the conversations that once occurred in a private residence among family are now more collaborative or communal in nature because of the desire to integrate health care facilities and personnel into the end of life process.

We now turn our attention to EOL communication, where we discuss the importance of these conversations from a family standpoint and from the perspective of PC practitioners. We then explore the intersection to illustrate how focusing on a tension common to patients, families, and practitioners privileges a powerful and productive starting point for making sense of and engaging in these pivotal discussions.

## 3. End of Life Conversations 

Though often deflected, conversations about end of life can provide a buffer against emotional isolation, ensure that one’s wishes are honored, and reduce possible miscommunication between medial teams, patients, and their families. Although beneficial, these conversations and even the word “death” is often evaded in communication surrounding end of life processes by both health care practitioners and lay people alike [4]. In its place, euphemistic expressions are favored as softer means to explain the harsh reality of death and dying [5]. The pervasiveness of avoiding talk about death and dying or using indirect or euphemistic language in U.S. American culture indicates a societal fear regarding end of life. In fact, death and the associated grieving process are often seen as a taboo topic and equated with a “disease” and something that one needs to quickly “get over” [6]. However, Western society’s avoidance or fear of talking openly about death does more harm than good for individuals actively dying and for the bereaved.

Although in many ways death is an individualized experience, a multitude of interpersonal others are affected by one’s EOL experience. Among other things, those we have relationships with provide us social support, inform the decisions we make regarding treatment, and ultimately survive us when we pass. A particularly salient group during EOL communication is our family.

### 3.1. Family End of Life Conversations 

Paramount to family scholarship is the recognition that family communication involves a set of interrelated and interdependent parts and in order to be fully understood families should be viewed as a system. Structure, organization, and transactional patterns within the family system influence individual behavior and communication [7]. To fully understand EOL within the family, researchers must examine the interplay of individual, relationship specific (e.g., brother-sister, father-daughter) and overall family-level influences that emerge in communication.

One promising approach to family EOL conversations would be to recognize the communication patterns within the family. Family Communication Patterns (FCP) theorizing has a longstanding history in family scholarship, and has provided researchers and practitioners a means of predicting and measuring the ways in which families communicate with one another [8]. FCP measures families in terms of conformity—the degree of homogeneity in attitudes, values, and beliefs amongst family members, and conversation—the degree of participation amongst family members in unrestrained interaction that covers a wide range of topics. Based on these two orientations, families can be categorized as either (a) *consensual*—those high in conversation and conformity; (b) *pluralistic*—those high in conversation but low in conformity; (c) *protective*—those low in conversation but high in conformity; or (d) *laissez-faire*—those low in conversation and low in conformity. More recent scholarship has moved away from a trait-like approach to FCP to investigate how the theory may be used to conceptualize family communication as state-like and that patterns vary depending on topic [9]. This research argues that patterns of family communication reorient depending on the topic being discussed, and that families do not necessarily possess static communicative orientations that encapsulate all potential topics that emerge during interaction. Regardless if viewing family communication orientations as static patterns or contingent upon topic, EOL conversations represent a complex communication context for families due to the variation in individual, relational, and family-level degrees of conformity and conversation. Considering family communication patterns toward end-of-life communication enables practitioners to gauge desired content and the degree or amount of communication desired by families.

Similar to how families orient around EOL conversations based on degrees of conformity and conversation, families also employ patterned privacy rules that shape their orientations to privacy choices [10]. EOL conversations in the family are ripe with dialectical tensions of privacy-disclosure as family members must negotiate what information is beneficial or detrimental to share and with whom to share it with. In order to manage information dissemination and ownership, families must construct and socialize its members to boundary rules.

Boundary rules provide guidelines to family members about sharing jointly owned information internally, as well as sharing information to those external of the family system. Successful boundary management requires families to recognize who is fastened into the privacy boundary, to what degree each individual has ownership rights to information, and what information can or cannot be leaked to parties outside of the family. Others [10] have identified three orientations to information boundaries exercised by families: (a) highly permeable—families that are prone to disclose information to one another and those outside of the family; (b) moderately permeable—families that are more judicious in their choices about who knows family information both internally and externally; and (c) lowly permeable—families where private information is highly restricted and where thick boundary lines reside around information. When managed successfully, privacy boundaries give families the ability to govern private information. However, and as is likely the case in EOL conversation, boundary turbulence often occurs in four specific ways.

First, family members disclose unexpected private information to certain individuals and ask those they tell to keep it confidential. An example of this would be in EOL conversations where a father tells his oldest child about his terminal illness, but requests the son keeps the information from his younger siblings. Second, family members may stumble across information they feel should be shared, but find themselves in situation where either disclosing or concealing the information would result in hurting another family member. For example, if the son in the previous example were to find documentation about his father’s diagnosis, the son may be at odds with addressing his father and concealing the information from his mother. Third, family members sometimes snoop and dig up information, but cannot act upon that information without admitting they’ve snooped. In this situation, the son may have shuffled through his dad’s dresser drawer and found documentation, but doesn’t want to admit he was rummaging through his dad’s private information. Finally, family members must make choices about what is best for them compared to the family as a whole or a specific family member. In this case, the father may be a struggling with sharing the information about his diagnosis to his family, or concealing it for fear of hurting his family.

Examining family communication orientations to conversation and conformity, as well as the ways in which families control and share information, augments how challenging EOL communication may be for families. In order to best understand EOL communication within the family, it is necessary to recognize the interdependent nature of families and the transactional patterns that construct family life. Given the complications of EOL conversations, families often turn to practitioners in championing their decisions during end of life. However, practitioners often find themselves navigating their own unique set of complications and tensions during EOL communication.

### 3.2. Practitioner End of Life Conversations 

Just as patients and their family members experience challenges in their EOL communication, practitioners too have to navigate various tensions. Palliative care clinicians, in particular, may find themselves in a precarious position. As medical professionals who frequently work with end of life patients and their families, they have been dubbed enforcers of death [11] because an initial purpose of the specialty was to predict the progression of an illness. Unfortunately, recent research [2] has revealed that this trend remains. That is, between the rise of the specialty and the incorporation of the communicatively oriented, patient-centered biosocial model of medicine, PC practitioners may be misunderstood and incur resistance from other medical professionals, while also shouldering the emotional weight of caring for patients with chronic and terminal illnesses.

Interprofessional communication among health care personnel of various disciplines is part of the challenge associated with practitioner EOL conversations. While collaboration among disparate medical areas, such as cardiology and palliative care, has been commonplace for decades and there is a hearty reliance on interdisciplinary healthcare teams, research continues to reveal misunderstandings, medical errors, and power struggles. Moreover, medical professionals tend to underestimate the importance of peers’ roles and or value in the process and therefore, then tend to discount their opinions or suggestions [12,13]. Researchers have long suggested that a core challenge associated with interprofessional communication is the fact that each medical specialty anchors its focus only on areas “which the profession has selected for observation and concern” [14] (p. 1799). This then suggests that medical professionals largely reject assumptions or points that are contrary to their conceptual framework. Put simply, it is not necessarily the inability to communicate clearly across medical disciplines that hinders interdisciplinary collaboration and promotes medical mistakes, but rather the fact that individual specialties may subscribe to different and competing goals.

Omilion-Hodges and Swords [2] found that PC practitioners frequently navigate two primary communication challenges: living-dying and practicing-advocating. These dialectics—tension between two opposing forces—present clear complications for practitioners as they work to administer holistic care inclusive of end of life conversations with patients and their important others. For instance, unlike other medical specialties, such as orthopedics or obstetrics and gynecology, PC practitioners often have to explain their purpose, persuade others of their value, and answer the question of “why do I need a doctor to help me die?” As such, PC practitioners have indicated feeling as though they have to serve as spokespeople for the profession so that they continue to secure resources ranging from marketing communication materials to easily accessible physical space and additional hires. In this sense, PC physicians are not only medical practitioners who are actively working to minimize the pain, fear and stress commonly associated with death and dying, but are also cheerleaders for the profession [2].

Another, and more cumbersome, communication challenge PC practitioners face is the perceived conflict between the focus of palliative care and that of the larger medical community: Living-dying. While palliative care is frequently administered in tandem with curative treatment, practitioners care for many near end of life and view death as natural. However, a steadfast commitment to prolonging life is embraced by virtually all other medical specialties. Considering that these foci can be interpreted as divergent, it is not surprising that many practitioners experience tension, confusion, or discomfort in EOL conversations. Moreover, research [2] has found that when attending physicians realize that extension of life is unlikely and recommend palliative care, they often forgo end of life conversations all together. PC professionals report that this likely stems from attending physicians who are in denial about a patient’s health status or are otherwise experiencing challenges in accepting that at patient is no longer a candidate for curative treatment. Avoidance of these sensitive conversations, therefore, is not confined within the dynamics of the family unit, but is also interwoven into the structure of the health system. The circumvention of the topic of death and dying by other medical professionals means that PC practitioners often find themselves in the position of having to explain to patients that they are not there to “build strength”, but rather because they are no longer responding to curative treatment. PC practitioners then serve as active and collaborative communicators for patients and their families in terms of discussing end of life concerns and wishes. Owing to this change and the raise of deaths that are occurring in organizational settings, it is important to consider how professionals and families communicate and cope during this decisive life experience. In this sense, neither practitioners nor patients and their families cope or communicate independently, but rather contemporary approaches to end of life have transformed this into an interdependent, relational, communicative process.

## 4. The Intersection of Family and Practitioner End of Life Communication

We have demonstrated how practitioners and families experience unique tensions surrounding conversations regarding EOL. However, we now narrow our focus to examine the communication challenges and foci common among patients, families, and health care practitioners. In particular, we will discuss the impact of communal coping and how commitment to quality of life, discussion of care vs. cure, and a resolute focus on communication is transforming EOL conversations.

Unlike individual coping and differentiated from social support, communal coping is largely defined by two criteria: appraisal and action [15]. In communal coping, appraisal and action suggest that members within a group co-own a specific stressor and experience it together. Put simply, “our problem, our responsibility” [15]. Applied to EOL communication, communal coping privileges a lens to explore how a patient, their family, and their PC team each assume a role and work together to assist in managing stressors. One particular stressor that is relevant across each of these groups is the tension between cure and care.

This tension can be a particularly challenging one to navigate for patients, families, and practitioners alike. While PC affirms death as a natural process, it does the same for life—so long as the patient indicates that he/she is satisfied with their quality of life. That is, for some curative treatment may mean severe sacrifices to daily life. In an editorial [16] a palliative care nurse shared the story of a young mother whose aggressive curative chemotherapy treatment came with feelings of impaired decision making, fogginess, pain and fatigue—and no noticeable change to the size of the growth. Therefore, after lengthy discussions with her family and PC team, the decision was made to reorient focus on care, rather than cure. This shift allowed the patient to spend her time as she wished, feeling like herself, and free from the painful side effects of the earlier intrusive curative treatment plan. Certainly, the decision made by the patient, her family, and her PC team is not a one size fits all solution. Moreover, it is likely that at times, the young mother, her spouse and their daughters, and perhaps even members of her PC team, did not necessarily want to accept care over cure. However, by taking a communal approach where each individual assumed responsibility for the illness and dedicated energy to actively considering how to address the disease, no one was left to navigate the EOL process unaided. Therefore, while the patient and her family may have been hesitant to support the cure to care shift, collectively with the woman’s PC team, they were able to discuss the probable progression of the disease, quality of life markers, and EOL fears. In this sense, even though the decision may not have been readily or immediately accepted by all, shared responsibility and concern for the patient’s wellbeing meant that pain control, fatigue management, and counseling would better support her needs.

This example helps to show how tightly coiled the tension between cure and care can be. Certainly the illustration above is one that tugs at the heart strings as the patient in question is youthful and has three young children. However, regardless of station in life, the natural inclination at a serious or terminal diagnosis is a focus on cure. In this traditional approach, typically the expert clinician orders a series of diagnostic tests, provides an opinion, prescribes a course of treatment, and uses an analysis pathway to compute the best possible outcome [17]. While often described as or perceived as in competition with one another, others [18] have argued that the two models—cure and care—are actually better considered as end points on one continuum. This conceptualization places more focus on the individual needs of the patient, where practitioners and the patient’s family collaborate for all members’ mutual benefit. This perspective intrinsically emphasizes the pivotal role that communication plays at end of life. In this sense, communication allows patients, families, and practitioners to engage in thoughtful conversations about goals and fears and to give and receive support. Since research indicates that initiating EOL discussions can be challenging for lay people and medical personnel alike, we offer two tangible communication suggestions for facilitating these conversations. Moreover, we provide ideas for how the suggestions may be utilized by a patient, his or her family, and or healthcare employees.

## 5. Takeaways: How to Initiate EOL Conversations 

While we have demonstrated some of the challenges of EOL communication, such as family dynamics or the tension between living-dying for PC practitioners, we have also begun to demonstrate how thoughtful communication can create opportunities for discussion. We now provide additional concrete communicative suggestions that may assist patients, family members, and practitioners in initiating and maintaining EOL dialogue. A constant commitment to thoughtful communication is key because patients expect compassion in others’ words and actions due to the emotional nature of end of life [19]. We now provide specific recommendations for the use of metaphors and key mindful communication practices to facilitate these complex conversations.

### 5.1. Metaphors 

Though EOL conversations are often avoided, talking about death is important for myriad reasons. While individuals may be hesitant to discuss death in direct terms, use of metaphors may ease the uncertainty surrounding these important conversations. That is, if we consider the abstract ways that individuals perceive death, such as a savior or thief, it can help us to make sense of how they ascribe meaning to death. Subsequently, this provides cues as how to initiate and maintain a conversation on the topic. Metaphors are powerful because they are central to how we reason and understand the world around us, particularly abstract, difficult subjects like death [20]. In this sense, metaphors can be interpreted as an opportunity to initiate a conversation about death, rather than an abstraction to be evaluated as either good or bad. Below we discuss common metaphors used to describe and make sense of death [21].

Individuals who may be more hesitant to discuss death and dying may consider death as inevitable or as the elephant in the room. Use of these metaphors accentuate the importance of the communication context in that some consider death as a complex and private topic and therefore are much more selective in terms of when or with whom they discuss it with. Moreover, because individuals who consider death in terms of an elephant in the room or inevitable often experience uncomfortable feelings that accompany these conversations and they see death as a boundary for communication. This reluctance may also stem from not knowing how to engage EOL conversations if we’re not certain of the beliefs of our conversational partner. In this sense, individuals who see death in this manner may wish to discuss EOL, but may be tentative because they don’t know how to broach the topic nor do they want to cause others’ discomfort.

If conceptualized as a mystery or a thief, it is a strong inclination that someone perceives EOL conversations as anxiety-ridden or personally painful and therefore, perceives them as something that should be avoided. When considered as a mystery, individuals expressly indicate a fear of the unknown or have challenges accepting the finality of death. Some who perceive death as a mystery may not necessarily view it as personally painful, but remain fixated on the unknown nature of what happens after death and what, if it exists, the afterlife might consist of. Others in this sample have suggested, particularly those without a particular spiritual or religious bend that fear of the afterlife prevents them from engaging in these conversations. Whereas the metaphor of death as a mystery is related to the unknown aspect of what happens after one dies, the use of a thief metaphor may show that the person in question has lost a number of loved ones or perceives death as stealing our time on earth. In this case, research [21] has indicated that individuals fostering these metaphors of death may avoid conversations because they serve as a painful reminder of feelings associated with the loss of a loved one.

While there are a number of metaphors linked with a reluctance to engage in EOL conversations, there are also several that may suggest a willingness or importance to discussing death. In an ongoing research project, Omilion-Hodges, Manning, and Swords [21] found that the largest proportion of participants viewed death in fairly positive terms, indicating an inclination to broach the subject. Within this camp, participants conceptualized death as natural, a savior, a motivator, and the unifier. The specific perspectives on death vary among these four metaphors, but they are all commonly linked by the notion that positive outcomes can be reached through open communication. Similar to the metaphor of death as inevitable, participants who frame death as natural see dying as a part of life. The distinction lies in the way that the individuals understand this perspective and their openness in communication as a result of their understanding. Relatedly, death as a savior, a motivator, and a unifier all rest of positive connotations of end of life suggesting that death may provide relief to loved ones who are in pain, can serve as a reminder of how precious and short life is, and that due to the rawness of death, boundaries are often broken down. An additional underlying theme is the idea that patients, families, and practitioners become co-owners of information about EOL processes including grieving and therefore, relationships may flourish because of the communal coping that occurs.

Death is not an easy topic for many, however, research indicates a growing willingness to engage in conversations about EOL. One way to learn another’s (or your own) perceptions of death, may be to ask them how they consider death. In learning how one refers to death, you may have a baseline understanding of how to initiate a conversation. Moreover, learning one’s metaphor may facilitate an opportunity to probe the metaphor to garner a deeper understanding of their perceptions of death. However, it is important to remember not to use the metaphor as a tool for binary evaluation—good or bad—but rather as a means to talk about EOL. Upon assessing someone’s conceptualization of death, use of mindful communication may then assist in maintaining a conversation.

### 5.2. Mindful Communication

In addition to employing common metaphors for death, use of mindful communication is also likely to increase the ease of EOL conversations. Mindful communication is an active process where communicators remaining attentive and engage in constant sensemaking of the content and context of the conversation. This allows individuals to employ reflective, authentic, and adaptive communication in any given situation. Considering this, mindful communication has been studied extensively within interpersonal and health communication settings and is linked with decreases in stress and professional isolation among physicians [22] and also with delegation of tasks and patient safety increases among nurses [23]. This practice has also been linked with success in communicating across cultures [24]. Most applicably, perhaps, mindful communication has been recommended as a vehicle for having EOL conversations and as a buffer against the emotional work of palliative care [1].

Omilion-Hodges and Swords [1] studied nationally-recognized palliative care practitioners to learn how industry leaders employed mindful communication to stave off burnout and deliver exemplary, patient-focused care. Ultimately, four key communication practices emerged that benefit patients, their family members, and the practitioners. The first key practice is to consider your audience. Knowing one’s audience means fundamentally rejecting a cookie-cutter approach to end of life communication. Bill, a hospice and palliative care physician and medical director, suggested that employing an authentic and individually-tailored approach to communication acted like a “relational slingshot” [1] (p. 331). To this effect, Bill found that use of mindful communication and a series of innocuous getting to know you questions, spurred the development of trusting, two-way relationships. Therefore, we stress, especially to practitioners, the importance of developing rapport with a patient and their loved ones before fixing focus on one’s medical history or care goals.

Relatedly, the second key practice resolved around asking questions, listening, and repeating the process. This mindful communication practice reminds practitioners and family members that the patient may need them to be a “person” before a “medical practitioner” or a “loved one” before a “patient advocate”. Therefore, during especially challenging communication encounters such as the delivery of a terminal diagnosis or offering an opinion in support of full-time or hospice care, practitioners and family members are encouraged to care for the patient holistically by affording them opportunities to ask questions, express disappointment, or emote sadness, anger, or fear. Further, by asking questions and listening, practitioners can gain insight into family desires regarding conversation initiation (i.e., practitioner or family) and the amount of communication they wish to engage in. The use of questions and engaged attention may help to facilitate this individual specific process. Similarly, palliative care leaders have found that mindful communication implicitly requires one to discard their scripts. As the third key practice, while scripts can be a helpful tool to spark ideas of how to breach EOL conversations, especially for new physicians or those in training, ultimately adherence to a pre-written narrative does not convey the authentic, adaptive communication required at EOL. This key practice ties in with the first in the sense that considering your audience often means that practitioner or family discomfort is relegated beneath patient needs. Certainly we do not recommend practitioners and family members to abandon self-care or individual needs, but rather remember to prioritize patient comfort at EOL which may include displaying genuine emotions such as disappointment or sadness.

The final key practice, and perhaps the most crucial, is to recognize your role. In this sense, PC practitioners emphasized the importance of remembering that a typical day in the office for them is a transformative event for patients and family members. To that end, the final key practice blends each of the previous to remind patients, families and practitioners that each is serving a crucial role. The patient, for example, may need to share or recant favorite memories in order to preserve them and promote generativity. Family members may need to ask questions or simply listen to their loved one’s stories, fears, or concerns. Finally, while practitioners are navigating organizational dynamics and stressors, it is essential that when they are communicating with patients and their important others, that they remain mindful and recognize that they a “main character” in each of their stories.

## 6. Discussion

In this essay, we have focused on how contemporary approaches to death and dying have prompted changes in the conversations that are occurring at EOL. While once confined to private residences, there has been a steep increase in demand for death to occur within organizational settings aligning patients, their important others, and medical professional as collaborators in the decision making and discussions that are part and parcel with EOL. Therefore, instead of the patient, family, or practitioner having to cope alone, current medical options—such as PC—meet society’s desire to utilize institutions at end of life and provide additional resources to help patients and their families to cope with this critical life experience.

Coping at end of life has also changed. While there is still a healthy level of stigma attached to discussing death and dying, at least in the Western context, interventions like Death over Dinner and the Conversation Project are helping people to talk about their wishes for EOL care. Sources such as these are empowering individuals to broach a once taboo topic and take the reins in terms of EOL planning. This same phenomenon is allowing families and practitioners to assume a “our problem, our responsibility” stance and work collaboratively to explore options, maintain quality of life, and when necessary, grieve [15]. Thus these transitions have prompted a healthier and more communicative approach to EOL where patients, families, and practitioners are active participants in designing and delivering a good death.

## 7. Conclusions

In conclusion, we have demonstrated how various tensions, especially the stress coiled within cure vs. care, are experienced by all members. While cure aligns more readily with the traditional biomedical model of care and cure with the more modern biosocial model, they both offer benefits to patients. Moreover, scholars [2] have continued to point out that there is room for the traditional cure model and the more nuanced care model in postmodern medicine. Considering this, communication becomes the key to determining specific, patient-tailored approaches to healthcare. Some tensions, such as care—cure, will likely never disappear, but communication can be the vehicle to spark and negotiate essential conversations and a means to cope.
